# Low-Frequency Ultrasound Enhances Bactericidal Activity of Antimicrobial Agents against *Klebsiella pneumoniae* Biofilm

**DOI:** 10.1155/2020/5916260

**Published:** 2020-01-03

**Authors:** Xu Liu, Jin Wang, Chun-xiao Weng, Rui Wang, Yun Cai

**Affiliations:** ^1^Center of Medicine Clinical Research, Department of Pharmacy, PLA General Hospital, Beijing, China; ^2^Savaid Medical School, University of Chinese Academy of Sciences, Beijing, China; ^3^Chiamery Medical Sciences Institute of Beijing, Beijing, China

## Abstract

*Klebsiella pneumoniae* biofilms on inserted devices have been proposed as one of the important factors for hospital-acquired infections, which cause increased resistance to currently used antibiotics. Therefore, it is urgently necessary to develop new treatments with more efficient bacterial clearance. In the present study, we aimed at investigating whether low-frequency ultrasound (LFU) could enhance the bactericidal activity of antimicrobial agents (meropenem (MEM), tigecycline (TGC), fosfomycin (FOM), amikacin (AMK), and colistin (COL)) against *K. pneumoniae* biofilm infection. *K. pneumoniae* biofilm was cultivated on the catheter *in vitro*. Synergistic effects were observed in groups of single ultrasound (S-LFU, 5 min) or multiple ultrasound (M-LFU, 5 min every 8 h (q8h)) in combination with MEM, TGC, and FOM. However, AMK and COL did not show the synergistic effect with either S-LFU or M-LFU. S-LFU in combination with FOM only significantly decreased bacterial counts right after ultrasound, while M-LFU could prolong the synergistic effect until 24 h. The results showed that LFU in combination with antimicrobial agents had a synergistic effect on *K. pneumoniae* biofilm, and M-LFU might extend the time of synergistic effect compared with S-LFU.

## 1. Introduction

The opportunistic pathogen, *Klebsiella pneumonia*, can trigger severe diseases, typically nosocomial infections, such as septicemia, pneumonia, urinary tract infection, and soft tissue infection [1]. Increasing evidence has proved the ability of *K. pneumoniae* to form biofilm, mostly on urinary catheters and tracheal tubes, and a mass of data have supported that such a behaviour plays a key role in the antibiotic resistance acquisition [2]. Compared with planktonic *K. pneumoniae*, the high-level resistance of *K. pneumoniae* biofilm has been confirmed on many antibiotics, such as piperacillin, meropenem (MEM), ciprofloxacin, netilmicin, and amikacin (AMK) [3]. In this context, antimicrobial combination therapy has become an option to treat infection with *K. pneumoniae* biofilm. In the absence of evidence-based treatment guidelines, clinicians are increasingly resorting to employ combination therapy for difficult-to-treat infections based on some weak but promising published data [4]. However, such combination regimes also bring higher risk of adverse events, leading to treatment failure, increased antibiotic use, and possible accelerated emergence of drug resistance [5]. Unorthodox combination of low-frequency ultrasound (LFU) and antimicrobial agents may bridge the gap in current treatment against biofilm infections. LFU has been reported in a series of studies as a promising method to enhance the antibiotic action on bacteria [6]. In the previous study, we have demonstrated the synergistic effect of LFU in combination with colistin (COL) or vancomycin against COL-resistant *Acinetobacter baumannii* biofilm. Currently, there is no published literature about studies of the synergistic effect of LFU in combination with antimicrobial agents against *K. pneumoniae* biofilm. In the present study, we, for the first time, investigated single ultrasound (S-LFU, 5 min) or multiple ultrasound (M-LFU, 5 min every 8 hours (q8h)) in combination with five different types of antimicrobial agents against *K. pneumoniae* biofilm. Meanwhile, the antibiofilm effects of S-LFU and M-LFU combinations were also compared.

## 2. Materials and Methods

### 2.1. Strains, Agents, and Antimicrobial Susceptibility Test

One *K. pneumoniae* strain was clinically isolated from the Academy of Military Medical Sciences and identified by the automated VUTEK 2 Compact System (BioMerieux, Marcy-l'Etoile, France) microbe analyser. *Pseudomonas aeruginosa* ATCC 27853 and *Escherichia coli* ATCC25922 were used as the quality control strains. MEM and tigecycline (TGC) were purchased from China Food and Drug Certification Institute. AMK and fosfomycin (FOM) were obtained from China National Institute for the Control of Pharmaceutical and Biological Products. COL was supplied by Sigma. According to CLSI guidelines, susceptibility test of five antimicrobial agents was performed by the broth microdilution method [7]. Briefly, 96-well plates were set up with antibiotics ranging from 0.00625 to 256 *μ*g/mL. Strain was grown on Mueller-Hinton agar (BD Difco, Franklin Lakes, NJ, USA), and then representative colonies were picked and suspended in Mueller-Hinton broth (MHB, BD Difco, Franklin Lakes, NJ, USA). Subsequently, 100 *μ*L bacterial suspension (1 × 10^5^ colony-forming units, CFU) was added to each well and then cultivated at 37°C. In addition, the maximum concentration in plasma, weight, and mechanism of five antibiotics were determined.

### 2.2. Cultivation of Biofilm

The biofilm was cultivated according to a previously described procedure [8]. Briefly, *K. pneumoniae* strain was incubated on catheter disks (diameter = 0.5 cm) in 24-well plates. Subsequently, 2 mL MHB and 100 *μ*L bacterial suspension (1.5 × 10^8^ CFU/mL) were added to each well, followed by incubation at 37°C for 3 days. MHB was refreshed every day.

### 2.3. LFU Apparatus

**Figure 1:**
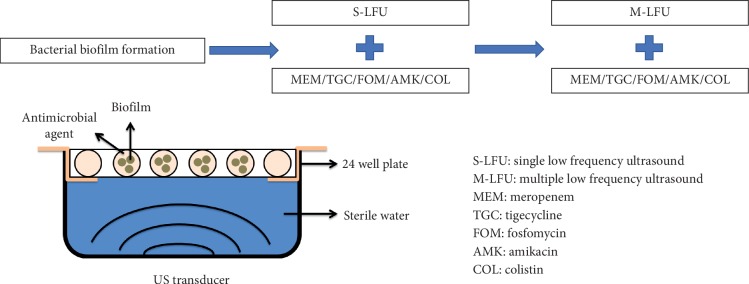
Diagram depicting using LFU and antimicrobial agents for the treatment of *K. pneumoniae* biofilms. Three catheter disks with biofilms were placed into each well of a 24-well plate containing 1 mL of antimicrobial agent solution. Sterile medium was added to the wells around the edge of the 24-well plate, serving as a negative control. LFU was transmitted through the bottom of the plate via sterile water. This study was to investigate the treatment effect of S-LFU or M-LFU in combination with five different types of antimicrobial agents.

LFU apparatus was provided by Beijing Nava Medical Technology. S-LFU and M-LFU had the same frequency and intensity, which were operated at 40 kHz with continuous irradiation at an intensity of 92.36 mW/cm^2^. S-LFU was operated for 5 min, and M-LFU was operated for 5 min (q8h) on *K. pneumonia* biofilm [8]. Three biofilm disks and 1 mL MHB in the presence of antimicrobial agents were added to each well of a 24-well plate. To avoid the effect of the holder, the same solution was added to the wells around the edge of the 24-well plate. The ultrasonic transducer was placed in a sterile water-filled ultrasonic bath, 7 cm below the 24-well plate [9]. There was no difference in water temperature before and after ultrasound treatment.  Figure 1 illustrates the diagram modified from a previous study [10].

### 2.4. Measurements of the Bactericidal Activity of S-LFU and M-LFU in combination with Five Antimicrobial Agents

A power intensity of 92.36 mW/cm^2^ was used in the present study, and the irradiation time was adjusted to 5 min for S-LFU and 5 min q8h for M-LFU. The biofilm disks were treated with MEM, AMK, TGC, and FOM at 4 × MIC or COL at 4 *μ*g/mL in the absence or presence of LFU [11]. After S-LFU or M-LFU treatment, the 24-well plates were cultivated at 37°C for 24 h. Right after ultrasound treatment or 24 h later, disks with biofilm were taken out. After the planktonic bacteria were washed off, the adherent bacteria on disks were collected by an ultrasonic cleaning bath for 10 min. The bacterial counts were determined by agar plates. Each treatment had six catheters. Bacterial counts were repeated three times.

### 2.5. Statistical Analysis

Statistical analysis was performed with GraphPad Prism software (San Diego, CA, USA). Data were presented as mean ± SD. Comparisons were carried out using one-way analysis of variance (ANOVA), followed by the Tukey–Kramer test for post hoc analysis. *P* < 0.05 was considered as statistically significant.

## 3. Results

### 3.1. Minimum Inhibitory Concentrations (MICs) and Related Information for Antimicrobial Agents

Table 1 summarizes the MICs and related drug information. *K. pneumoniae* strain was susceptible to MEM, AMK, and TGC, but resistant to FOM and COL.

### 3.2. Activity of S-LFU in combination with Antimicrobial Agents against *K. pneumoniae* Biofilm

**Figure 2:**
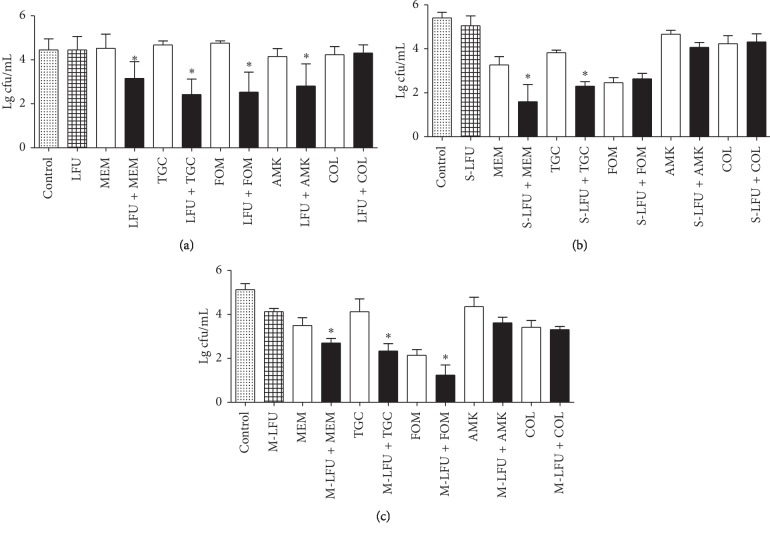
Synergistic effect of S-LFU or M-LFU in combination with antimicrobial agents against *K. pneumoniae* biofilms. (a) Bacterial counts in the biofilm of S-LFU and antimicrobial agents right after ultrasound treatment. (b) Bacterial counts in the biofilm of S-LFU and antimicrobial agents at 24 h. (c) Bacterial counts in the biofilm of M-LFU and antimicrobial agents at 24 h. S-LFU: single low-frequency ultrasound; M-LFU: multiple low-frequency ultrasound; MEM: meropenem; TGC: tigecycline; FOM: fosfomycin; AMK: amikacin; COL: colistin. ^*∗*^*P* < 0.05, as compared with the control, S-LFU or M-LFU, and antimicrobial agent treatment without S-LFU or M-LFU groups.

Figures 2(a) and 2(b) show that the *K. pneumoniae* biofilm disks were treated with S-LFU in combination with MEM, AMK, TGC, and FOM at 4 × MIC, or COL at 4 *μ*g/mL. Viable bacterial counts in biofilms were determined right after ultrasound treatment and 24 h later. Right after ultrasound treatment, bacterial counts were significantly decreased in S-LFU plus MEM, TGC, FOM, or AMK groups compared with the individual drug groups, while the synergistic effect was retained until 24 h only in the S-LFU plus MEM or TGC group. At 24 h, viable bacterial counts were significantly decreased in all groups compared with the control group, except for AMK and S-LFU alone groups. No decrease in viable bacterial counts was observed in S-LFU and COL combination group right after ultrasound treatment and 24 h later.

### 3.3. Activity of M-LFU in combination with Antimicrobial Agents against *K. pneumoniae* Biofilm

Figure 2(c) illustrates that the *K. pneumoniae* biofilm disks were treated with M-LFU in combination with MEM, AMK, TGC, and FOM at 4×MIC, or COL at 4 *μ*g/mL. Compared with individual drug groups, viable bacterial counts were significantly decreased in all M-LFU combination groups, except for M-LFU plus AMK or COL group. Similar to S-LFU plus antimicrobial agent groups, viable bacterial counts were significantly decreased in all groups compared with the control group, except for the AMK alone group.

## 4. Discussion

*K. pneumoniae* is able to form biofilms, and these adherent cells are often embedded within a self-produced matrix of extracellular polymeric substance. Biofilms are most notorious for high-level resistance to antibiotics [18]. Therapies for biofilm infections remain very difficult, and successful cases are quite rare. LFU is a promising method to treat biofilm infections due to its advantages, such as beam directivity and capability of treating deep tissue targets without tissue damage [19]. To the best of our knowledge, we, for the first time, investigated the effects of LFU in combination with antimicrobial agents against *K. pneumoniae* biofilm *in vitro.*

Figure 2(a) shows that all groups of S-LFU in combination with antimicrobial agents had the antibiofilm effects right after ultrasound treatment, except for S-LFU plus COL group. LFU at the physiotherapy level can enhance the transfer efficiency of drugs, leading to improved lethal effects of antimicrobial agents on drug-resistant bacteria or biofilm [9,20]. Investigation regarding the effectiveness of antibacterial substances in combination with ultrasonic therapy has now become a research hotspot in the treatment of biofilm infections, and certain preliminary clinical studies have already been performed [21]. The exact mechanism of synergy remains largely unexplored. Currently, most studies suggest that cavitation is the main responsible cause for the synergistic effect. Liquid medium can form microbubbles, which may act on biofilms and increase its permeability to antimicrobial agents or even kill bacteria in biofilm [22–24]. However, for *K. pneumoniae*, many studies have demonstrated that the limited penetration of antibiotic molecules through the biofilm matrix is not the main reason for the increased resistance, but rather, the slow growth rate in the center of biofilm is [1]. LFU treatment can promote more oxygen and nutrition into biofilm, which accelerates bacterial growth and restores their susceptibility to antibiotics [19]. This may be a factor regulating the synergistic effect of LFU and antibiotics against *K. pneumoniae* biofilm.

Many factors, such as intensity, frequency, irradiation time, and duty cycle, can affect the activity of LFU against biofilms [25]. The type of antimicrobial agent is also a factor affecting the synergy between LFU and antimicrobial agent. For *Enterobacter aerogenes*, gentamicin and kanamycin in combination with LFU show better antibacterial effect than streptomycin [26]. The potent synergistic mechanism may not only improve biofilm permeability or accelerate bacteria growth, but also affect antibacterial mechanism of antimicrobial agents. In this study, we investigated the synergistic effects of LFU and five antimicrobial agents, including MEM, TGC, FOM, AMK, and COL. Those drugs were selected based on the recommended treatment drugs for *K. pneumoniae* in a Chinese consensus statement [27]. Table 1 shows that the antibacterial mechanisms of those drugs were different. At 24 h, the synergistic antibiofilm effects of S-LFU plus AMK or FOM disappeared compared with those right after ultrasound treatment (Figure 2(b)). Only S-LFU in combination with MEM or TGC could significantly decrease the bacterial counts compared with drug alone. The relationship of antimicrobial agent mechanism and synergistic effect remains unclear and needs to be clarified in future study.

Figure 2(c) shows that the bactericidal effect was observed from the M-LFU alone group. M-LFU in combination with MEM, TGC, or FOM had a synergistic effect, as the bacterial counts were significantly decreased in M-LFU combination groups compared with drug alone groups. However, no synergy was observed in S-LFU plus FOM at 24 h. These results were similar to our previous study [10]. M-LFU in combination with vancomycin has a synergistic effect against MRSA biofilm, while S-LFU does not show such effect. This study also proved that M-LFU had distinct potential to facilitate antibiotics and obtain the better effect than S-LFU.

Significantly decreased bacterial counts were observed in MEM, TGC, or FOM alone at 4 MIC, indicating that these antimicrobial agents had antibiofilm effect on *K. pneumoniae* biofilm, while no antibiofilm effect was observed in AMK or COL alone group. For COL, the concentration used was too low to exert antibiofilm effect. Moreover, a COL-resistant *K. pneumoniae* strain was used in this study. We intended to use LFU to promote the COL antibiofilm effect. However, the result was negative. Interestingly, Sato et al. have indicated that COL at sub-MIC (1/2 or 1/4 MIC) can promote biofilm formation of *Acinetobacter baumannii*. It may depend on efflux pumps and biofilm-related genes regulated by COL [28]. This might be a reason why synergistic effect was not observed in the LFU plus COL group. COL at sub-MIC showed antibiofilm effects, while it also promoted biofilm formation of *K. pneumonia.* Such confusing findings should be clarified in the future study. AMK is a hydrophilic drug with the lowest log*P* (−8.6, data from ChemAxon, the logarithm of the octanol/water partition coefficient) among the five antimicrobial agents tested in the study. Although LFU could damage the bacterial biofilm, AMK might be still difficult to penetrate biofilm and cell membrane due to its high hydrophilicity. The biofilm consists of a region of densely packed cells without prominent pores, and cell membrane is mainly composed of hydrophobic phospholipid [29]. Besides due to the incomplete antimicrobial penetration, bacteria in biofilm generally are more resistant than those in planktonic state. The low metabolic state of bacteria may be attributed to such resistance [30]. Anderl et al. [31] have demonstrated that ciprofloxacin can penetrate *K. pneumoniae* biofilm but cannot kill the bacteria. In the present study, a small amount of AMK that penetrated through the biofilm was not able to kill the bacteria at low metabolic state.

Although seldom clinical trials have tested the synergy of LFU in combination with antibiotics in patients, LFU, as a noninvasive treatment, still remains a promising method against biofilm infection. For example, the high incidence of biofilm infections associated with medical devices, such as catheters or implants, is a difficult problem in clinical practice [32,33]. Scientists incorporate antibiotics into the devices or materials, which target the sites where biofilm formation is likely to occur, to inhibit biofilm formation. LFU could not only promote antibiotic release from implanted material, but also synergistically combine with antibacterial agents to achieve better therapeutic effects. Besides, combination therapy of LFU and antimicrobial agents may be beneficial for chronic wound infection, which is also a typical biofilm infection [19].

Collectively, we found that LFU, either S-LFU or M-LFU, in combination with antimicrobial agents had a synergistic effect. The synergistic antibiofilm effect of M-LFU could last longer compared with S-LFU in combination with antimicrobial agents. The antibacterial mechanism might affect the synergy. The *in vitro* data presented here suggested that further investigations should be performed on the mechanism involved in the synergistic effect, as well as its applications *in vivo.*

## Figures and Tables

**Table 1:** MICs and drug information of antimicrobial agents against *K. pneumoniae*.

Antimicrobial agent	MIC (*μ*g/mL)	MIC interpretive criterion	Maximum concentration in plasma	Weight^a^	Mechanism
Meropenem	0.0625	≤1 S	49 (39–58) *μ*g/mL [12] (1 g intravenous infusion)	383.46	A bactericide for the bacterial breeding season
		2 I			Multiplication stage bactericide inhibition of cell wall synthesis by binding to penicillin-binding protein [12]
		≥4			

Tigecycline	0.5	≤4 S	1.45(22%) *μ*g/mL [13] (100 mg intravenous infusion)	585.64	Bacteriostatic agent
		8 I			Inhibition protein translation by binding to the 30S ribosomal subunit and blocking the entry of aminoacyl tRNA molecules into the A site of the ribosome [13]
		≥16 R			

Fosfomycin	256	≤64 S	370 ± 61.9 *μ*g/mL (8 g intravenous infusion) [14]	138.06	A bactericide for the bacterial breeding season
		128 I			Inhibition of peptidoglycan synthesis in the bacterial cell wall [14]
		≥256 R			

Amikacin	1	≤16 S	90.6 (71.7–105.3) *μ*g/mL (25 mg/kg intravenous infusion) [15]	585.60	Bactericide for rest period disruption and inhibition of protein synthesis by binding to the 30S ribosomal subunit [16]
		32 I			
		≥64 R			

Colistin	32	≤2 S	12.8 ± 6.2 μg/mL in sputum (4 million IU nebulized) [17]	1253.51	Bactericide for rest period
		>2 R			Surface active agent which penetrates and disrupts the bacterial cell membrane [17]

Concentrations are showed as mean ± SD, median (interquartile range), or mean (CV%). MIC interpretive criteria of meropenem, amikacin, tigecycline, and fosfomycin were based on CLSI. MIC interpretive criterion of colistin was based on EUCAST. ^a^Data from DrugBank.

## Data Availability

The authors confirm that the data supporting the findings of this study are available within the article.
